# Three New Triterpenoids from European Mushroom *Tricholoma terreum*

**DOI:** 10.1007/s13659-015-0071-5

**Published:** 2015-09-09

**Authors:** Tao Feng, Juan He, Hong-Lian Ai, Rong Huang, Zheng-Hui Li, Ji-Kai Liu

**Affiliations:** College of Pharmacy, South-Central University for Nationalities, Wuhan, 430074 China; College of Life Sciences, South-Central University for Nationalities, Wuhan, 430074 China

**Keywords:** *Tricholoma terreum*, Triterpenoids, Cytotoxicity

## Abstract

Three rare triterpenoids, saponaceolides Q–S (**1**–**3**), have been isolated from fruiting bodies of the mushroom *Tricholoma terreum*. Their structures were characterized based on extensive spectroscopic data. Compound **1** showed certain cytotoxicities against four human tumor cell lines.

**Electronic supplementary material:**

The online version of this article (doi:10.1007/s13659-015-0071-5) contains supplementary material, which is available to authorized users.

## Introduction

**Fig. 1:**
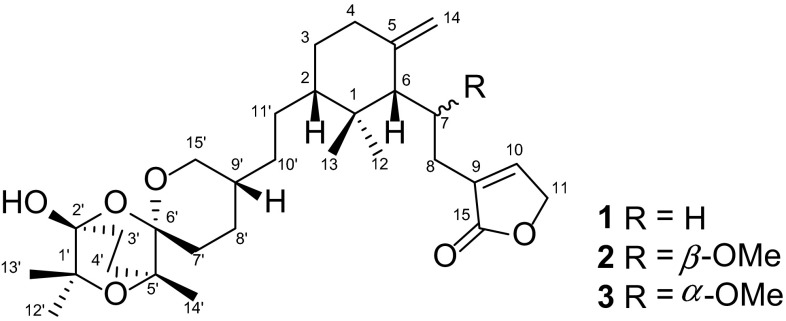
Structures of compounds **1**–**3**

Our previous work has identified *Tricholoma terreum* as a hitherto unknown poisonous European mushroom [1]. From which fifteen new triterpenoids terreolides A**–**F and saponaceolides H**–**P have been isolated. Terreolides A**–**F possessed novel frameworks, while saponaceolides B and M were the main toxins in the mushroom. The structural diversity, as well as important bioactivity discovery, prompted us to make a further study on this mushroom. According to an investigation on chloroform extract of *T. Terreum* collected in Arcachon in southwestern France, three new triterpenoids, saponaceolides Q–S (**1**–**3**), have been obtained (Fig. 1). Their structures were established by extensive spectroscopic methods. Compounds **1**–**3** were evaluated for their cytotoxicities against five human cancer cell lines.

## Results and Discussion

**Fig. 2:**
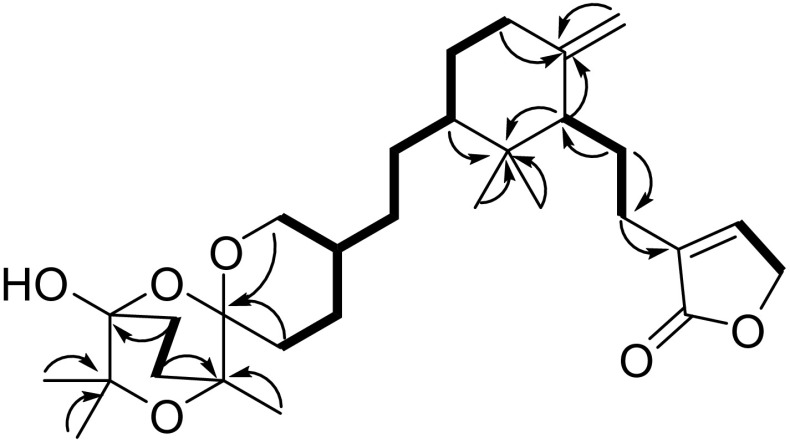
Key 2D NMR correlations of saponaceolide R (**2**)

Compound **1** was isolated as a colorless oil. The HRESIMS data (*m/z* 525.3193 [M + Na]^+^) indicated the molecular formula C_30_H_46_O_6_, requiring eight degrees of unsaturation. The IR absorption bands at 3436 and 1723 cm^−1^ suggested the presence of hydroxy and carbonyl groups, respectively. In the ^1^H NMR spectrum (Table 1), five singlets from *δ*_H_ 0.54 to 1.29 were readily identified signals for five methyls, while a singlet at *δ*_H_ 7.11 (1H, br s, H-11) revealed the existence of a double bond that might have an *α*,*β*-unsaturated keto moiety. The ^13^C NMR and DEPT spectra displayed thirty carbon resonances that was classified into five CH_3_, thirteen CH_2_, four CH, and eight C, as shown in Table 2. These data displayed high similarities to those of saponaceolide O, a triterpenoid isolated from the same mushroom by Yin et al. [1]. The key difference in **1** was that C-7 was identified as a methylene (*δ*_H_ 1.82 and 1.67, m; *δ*_C_ 23.1) rather than a carbonyl carbon in saponaceolide O, which was supported by ^1^H–^1^H COSY fragments of H-7 with *δ*_H_ 1.68 (1H, m, H-6) and 2.48 (1H, m, H-8a), as well as HMBC correlations from H-7 to *δ*_C_ 53.5 (d, C-6) and 24.7 (t, C-8) (Fig. 2). Analyses of 2D NMR data suggested that the other parts of **1** were the same to those of saponaceolide O (Fig. 2) [1]. Compound **1** was, therefore, elucidated as shown in Fig. 1 and named saponaceolide Q.

**Table 1:** ^1^H NMR data for compounds **1**–**3** (*δ* in ppm, *J* in Hz)

Entry	**1**	**2**	**3**
2	1.09 overlapped	1.06, overlapped	1.15 overlapped
3	1.78 m; 1.09 m	1.76 m; 1.07 m	1.79 m; 1.13 m
4	2.32 m; 1.92 m	2.29 m; 1.88 m	2.34 m; 1.98 m
6	1.68 m	1.60 m	2.13 m
7	1.82 m; 1.67 m	4.13 dd (7.6, 5.2)	4.10 d (10.4)
8	2.48 m; 2.14 m	2.01 m	1.86 m; 1.63 m
10	7.11 br s	7.35 br s	7.33 br s
11	4.78 br s	4.84 d (6.0)	4.84 d (17.0)
12	0.99 s	0.94, s	0.99, s
13	0.54 s	0.53, s	0.52, s
14	4.88 br s; 4.59 br s	4.87, br s; 4.64, br s	4.94, br s; 4.88, br s
3′	2.00 m; 1.87 m	2.00 m; 1.87 m	2.00 m; 1.88 m
4′	2.16 m; 1.68 m	2.16 m; 1.67	2.17 m; 1.67
7′	1.97 m; 1.51 m	1.96 m; 1.50 m	1.97 m; 1.51 m
8′	1.67 m	1.65 m	1.67 m
9′	1.48 m	1.47 m	1.49 m
10′	1.26 m; 1.03 m	1.23 m; 1.01 m	1.26 m; 1.04 m
11′	1.55 m; 0.81 m	1.53 m; 0.79 m	1.56 m; 0.82 m
12′	1.29 s	1.29 s	1.29 s
13′	1.21 s	1.21 s	1.22 s
14′	1.09 s	1.09 s	1.10 s
15′	3.69 dd (11.0, 10.9) 3.60 dd (11.0, 4.3)	3.68 dd (11.0, 10.8) 3.58 dd (11.0, 4.2)	3.69 dd (11.3, 10.8) 3.60 dd (11.3, 4.0)
MeO-		3.30 s	3.30 s

Data (*δ*) were measured in CDCl_3_. The assignments were based on DEPT, ^1^H–^1^H COSY, HSQC, and HMBC experiments

**Table 2:** ^13^C NMR data for compounds **1**–**3** (*δ* in ppm)

No.	**1**	**2**	**3**
1	39.8 C	39.7 C	39.5 C
2	48.0 CH	47.9 CH	48.1 CH
3	30.3 CH_2_	29.1 CH_2_	30.5 CH_2_
4	37.3 CH_2_	37.2 CH_2_	37.5 CH_2_
5	148.0 C	148.8 C	148.1 C
6	53.5 CH	49.2 CH	49.3 CH
7	23.1 CH_2_	75.2 CH	75.4 CH
8	24.7 CH_2_	30.5 CH_2_	32.0 CH_2_
9	134.8 C	135.0 C	136.5 C
10	143.8 CH	146.8 CH	144.9 CH
11	70.1 CH_2_	70.2 CH_2_	70.6 CH_2_
12	26.5 CH_3_	26.4 CH_3_	26.6 CH_3_
13	14.9 CH_3_	14.9 CH_3_	15.3 CH_3_
14	106.7 CH_2_	106.5 CH_2_	107.9 CH_2_
15	174.4 C	172.8 C	173.2 C
1′	77.5 C	77.5 C	77.7 C
2′	96.6 C	96.6 C	96.8 C
3′	27.9 CH_2_	27.9 CH_2_	28.1CH_2_
4′	28.5 CH_2_	28.5 CH_2_	28.4 CH_2_
5′	72.8 C	72.8 C	73.0 C
6′	101.4 C	101.2 C	101.5 C
7′	29.2 CH_2_	29.2 CH_2_	29.5 CH_2_
8′	24.8 CH_2_	24.8 CH_2_	25.1 CH_2_
9′	35.7 CH	35.6 CH	35.9 CH
10′	31.7 CH_2_	31.6 CH_2_	31.9 CH_2_
11′	27.6 CH_2_	27.6 CH_2_	28.1 CH_2_
12′	25.9 CH_3_	25.9 CH_3_	26.1 CH_3_
13′	22.4 CH_3_	22.4 CH_3_	22.6 CH_3_
14′	20.9 CH_3_	20.9 CH_3_	21.1 CH_3_
15′	65.8 CH_2_	65.9 CH_2_	66.2 CH_2_
MeO-		57.2 CH_3_	58.4 CH_3_

Data (*δ*) were measured in CDCl_3_. The assignments were based on DEPT, ^1^H–^1^H COSY, HSQC, and HMBC experiments

Compound **2** was isolated as a colorless oil. The HRESIMS ion peak at *m/z* 555.3290 ([M + Na]^+^) (calcd. 555.3292) indicated the molecular formula C_31_H_48_O_7_, requiring eight degrees of unsaturation. The ^1^H and ^13^C NMR spectra displayed similar patterns to those of **1** except for the signals at *δ*_H_ 4.13 (1H, dd, *J* = 7.6, 5.2 Hz, H-7) and 3.30 (3H, s, MeO-) and *δ*_C_ 75.2 (d, C-7) and 57.2 (q, MeO-), suggesting the existence of a methoxy substituent. The HMBC correlation from *δ*_H_ 3.30 (3H, s, MeO-) to C-7 indicated that a methoxy group placed at C-7, which was also supported by ^1^H–^1^H COSY correlations of H-7 with *δ*_H_ 1.60 (1H, m, H-6) and 2.01 (2H, m, H-8). Detailed analyses of 2D NMR data suggested that other parts of **2** were the same to those of **1**. The coupling constant of H-7 (*J* = 7.6, 5.2 Hz) suggested an *S* configuration of C-7 referring to the data of the literature [1–4]. Therefore, compound **2** was determined to be saponaceolide R.

The 1D NMR (Tables 1 and 2) and HRESIMS data of **3** were almost the same to those of **2**, which informed that **3** might have the same framework to that of **2**. Detailed analyses of 2D NMR data suggested that **3** did possess the same planar structure to that of **2**. However, tiny changes of NMR data of CH-6, CH-7, and CH_2_-8 suggested that the stereoconfiguration of C-7 was different from that of **2**. The coupling constant of H-7 (d, *J* = 10.4 Hz) was also significantly different to those reported previously (less than 8 Hz) [1–4], which allowed an R configuration of C-7 in **3**, that was also in agreement with those described in the literature [3]. Therefore, compound **3** was established and named saponaceolide S.

Many triterpenoids in this type, such as saponaceolides B, E, and F, have been reported to possess cytotoxicities to several cancer cell lines [2–4]. Compounds **1**–**3** were, therefore, evaluated for their cytotoxicities to five human cancer cell lines. As a result, compound **1** showed moderate activities as shown in Table 3.

**Table 3:** Cytotoxicities of compounds **1**–**3** (IC_50_, μM)

Entry	HL-60	SMMC-7721	A-549	MCF-7	SW480
**1**	12.2	19.3	>40	12.2	1.4
**2**	>40	>40	>40	>40	>40
**3**	>40	>40	>40	>40	>40
Cisplatin	2.4	11.2	17.6	18.7	14.9

## Experimental

### General Experimental Procedures

Optical rotations were measured on a Jasco-P-1020 polarimeter. IR spectra were obtained using a Bruker Tensor 27 FT-IR spectrometer with KBr pellets. NMR spectra were acquired with instrument of a Bruker DRX-600 with tetramethylsilane (TMS) used as an internal standard at room temperature. HRESIMS were recorded on an API QSTAR pulsar spectrometer. Silica gel (200–300 mesh), Sephadex LH-20 and RP-18 gel (20–45 µm) were used for column chromatography (CC). Fractions were monitored by thin layer chromatography and spots were visualized by heating silica gel plates immersed in H_2_SO_4_ in EtOH, in combination with the Agilent 1200 series HPLC system (Eclipse XDB-C18 column, 5 μm, 4.6 × 150 mm).

### Mushroom Material

Wild mushrooms, *T. terreum*, were collected from Arcachon in southwestern France in December 2012 and identified by Prof. Zhu-Liang Yang of Kunming Institute of Botany, Chinese Academy of Sciences. A specimen (No. KIB20121205.2) was deposited at the Kunming Institute of Botany, Chinese Academy of Sciences. For details of this mushroom please see that reported previously [1].

### Extraction and Isolation

The fresh fruiting bodies of *T. Terreum* (3 kg) were extracted with chloroform (24 h × 3), and then partitioned with water (1:1). Finally, a chloroform extract (12 g) was obtained, which was submitted to silica gel CC using petroleum-acetone (from 1:0 to 0:1) to give six fractions (A–F). Fraction B (1.8 g) was separated by reverse-phased CC eluted with gradient mixture of MeOH and H_2_O (30:70–100:0, v/v) to afford five sub-fractions (B1–B5). Fraction B2 (32 mg) was purified by Sephadex LH-20 (MeOH) to give compound **1** (2.2 mg), while fraction B4 (57 mg) was also purified by Sephadex LH-20 (MeOH) to give compounds **2** (2.3 mg) and **3** (1.3 mg).

#### Saponaceolide Q (**1**)

Colorless oil, $\it \upalpha_{\text{D}}^{\text{23}}$ + 7.2 (*c* 0.12 MeOH); IR (KBr) ν_max_ 3436, 2937, 1723, 1448, 1367, 1201, 1068, 991 cm^−1^; for ^1^H (600 MHz) and ^13^C NMR (150 MHz) data (CDCl_3_), see Tables 1 and 2, respectively; HRESIMS: *m/z* 525.3193 (calcd for C_30_H_46_O_6_Na, [M + Na]^+^, 525.3187).

#### Saponaceolide R (**2**)

Colorless oil, $\it \upalpha_{\text{D}}^{\text{23}}$ + 19.7 (*c* 0.10 MeOH); IR (KBr) ν_max_ 3443, 2926, 1726, 1457, 1381, 1065, 998 cm^−1^; for ^1^H (600 MHz) and ^13^C NMR (150 MHz) data (CDCl_3_), see Tables 1 and 2, respectively; HRESIMS: *m/z* 555.3290 (calcd for C_31_H_48_O_7_Na, [M + Na]^+^, 555.3292).

#### Saponaceolide S (**3**)

Colorless oil, $\it \upalpha_{\text{D}}^{\text{23}}$ + 13.7 (*c* 0.11 MeOH); IR (KBr) ν_max_ 3441, 2926, 1724, 1452, 1382, 1120, 997 cm^−1^; for ^1^H (600 MHz) and ^13^C NMR (150 MHz) data (CDCl_3_), see Tables 1 and 2, respectively; HRESIMS: *m/z* 555.3290 (calcd for C_31_H_48_O_7_Na, [M + Na]^+^, 555.3292).

### Cytotoxicity Assay

Human myeloid leukemia HL-60, hepatocellular carcinoma SMMC-7721, lung cancer A-549 cells, breast cancer MCF-7 and colon cancer SW480 cell lines were used in the cytoxic assay. All cell lines were cultured in RPMI-1640 or DMEM medium (Hyclone, USA), supplemented with 10 % fetal bovine serum (Hyclone, USA) in 5 % CO_2_ at 37 °C. The cytotoxicity assay was performed according to the MTT (3-(4,5-dimethylthiazol-2-yl)-2,5-diphenyl tetrazolium bromide) method in 96-well microplates [5]. Cisplatin was used as a positive control.

## Electronic supplementary material

Supplementary material 1 (PDF 8569 kb)
