# Supplementary material for: Three New Triterpenoids from European Mushroom Tricholoma terreum
Source: Nat Prod Bioprospect. 2015 Sep 9;5(4):205–8. doi: 10.1007/s13659-015-0071-5 (PMC4567990; doi:10.1007/s13659-015-0071-5)
Supplement: Supplementary file 1 — Supplementary material 1 (PDF 8569 kb) [file 13659_2015_71_MOESM1_ESM.pdf]

Supporting Information

Three New Triterpenoids from European Mushroom *Trcholoma terreum*

Tao Feng[a], Juan He[a], Hong-Lian Ai[b], Rong Huang[a], Zheng-Hui Li[a,]*, Ji -Kai Liu[a,]*

[a] *College of Pharmacy, South-Central University for Nationalities, Wuhan, 430074, China*

[b] *College of Life Sciences, South-Central University for Nationalities, Wuhan, 430074, China*

** Corresponding author: lizhenghui@mail.kib.ac.cn; jkliu@mail.kib.ac.cn*

NMR and HRESIMS for Compounds 1–**3**

NMR and HRESIMS for compound 1

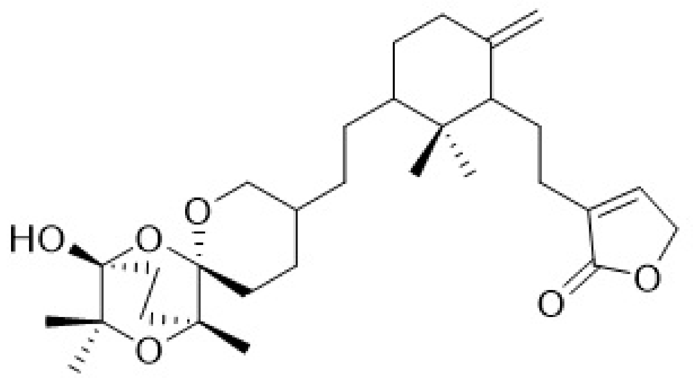

Saponaceolide Q

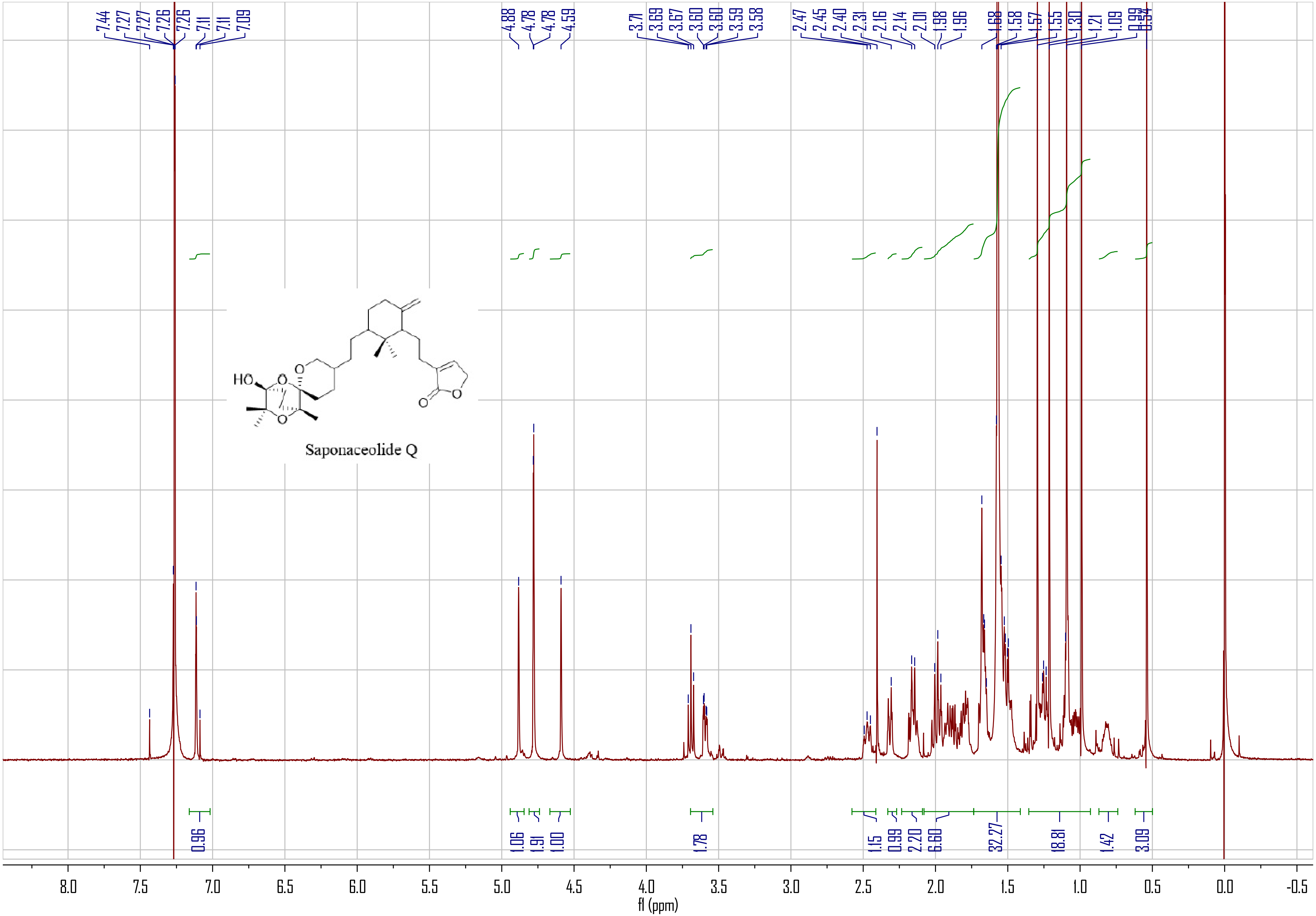
7.44
7.27
7.27
7.26
7.26
7.11
7.11
7.09
4.88
4.78
4.78
4.59
3.71
3.69
3.67
3.60
3.60
3.59
3.58
2.47
2.45
2.40
2.31
2.16
2.14
2.01
1.98
1.96
1.68
1.58
1.57
1.55
1.30
1.21
1.09
0.99
0.54
HO
O
O
O
O
O
Saponaceolide Q
0.96
1.06
1.91
1.00
1.78
1.15
0.99
2.20
6.60
32.27
18.81
1.42
3.09
8.0
7.5
7.0
6.5
6.0
5.5
5.0
4.5
4.0
3.5
3.0
2.5
2.0
1.5
1.0
0.5
0.0
-0.5
fl (ppm)

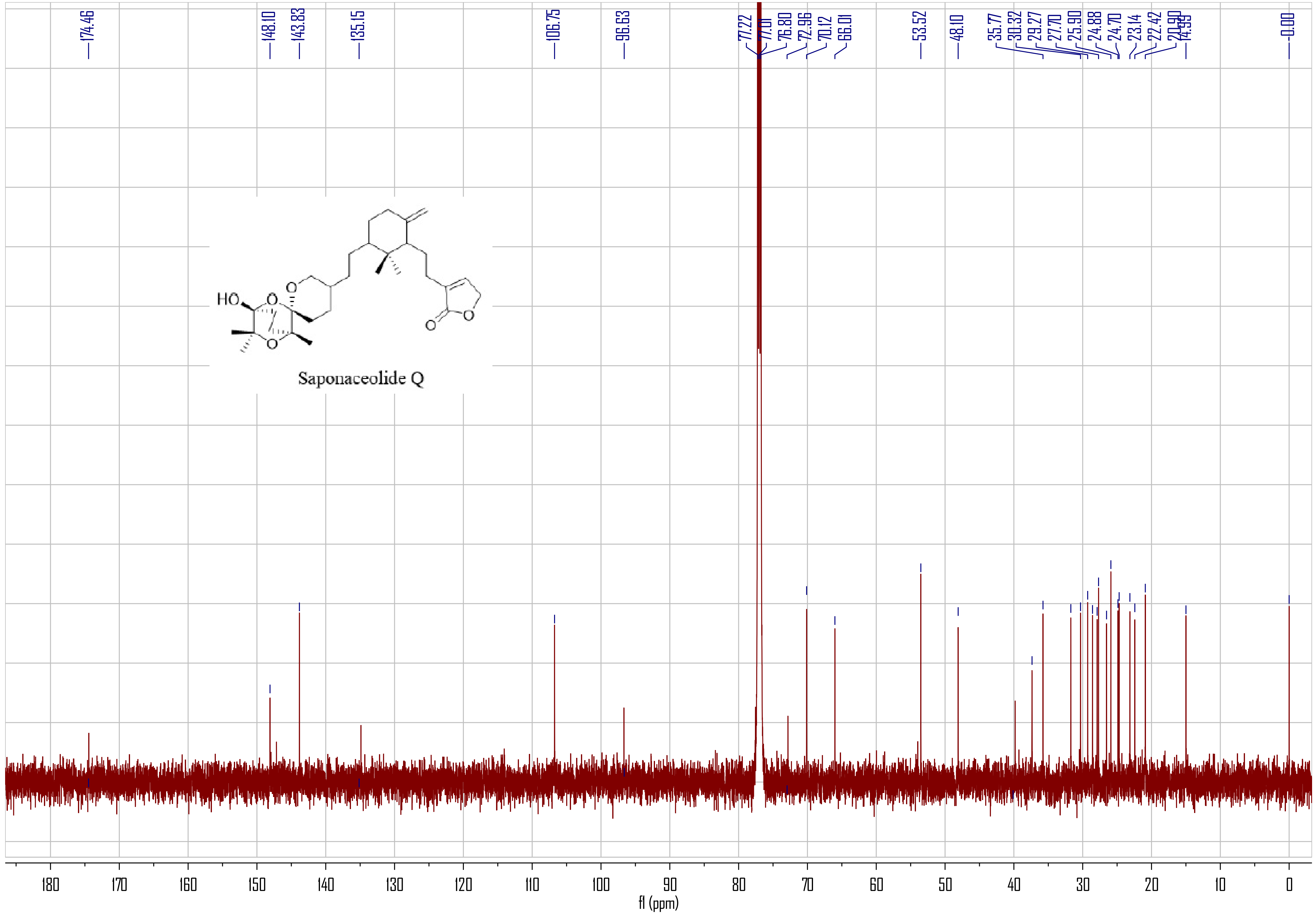

174.46
148.10
143.83
135.15
106.75
96.63
77.22
77.01
76.80
72.96
70.12
66.01
53.52
48.10
35.77
30.32
29.27
27.70
25.90
24.88
24.70
23.14
22.42
20.90
0.00
HO
O
Saponaceolide Q
180
170
160
150
140
130
120
110
100
90
80
70
60
50
40
30
20
10
0
fl (ppm)

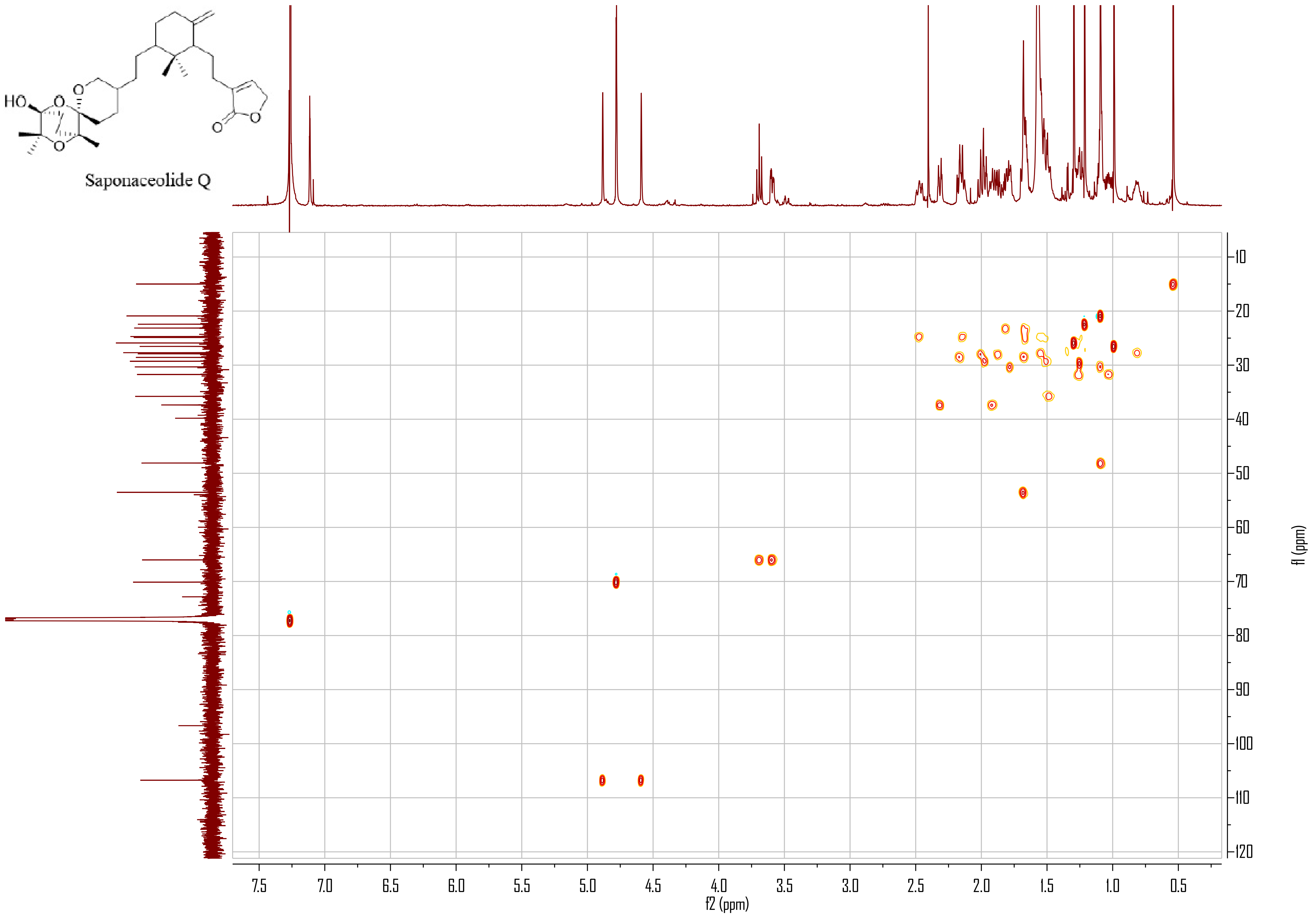

HO
O
O
O
O
O
Saponaceolide Q
f2 (ppm)
f1 (ppm)

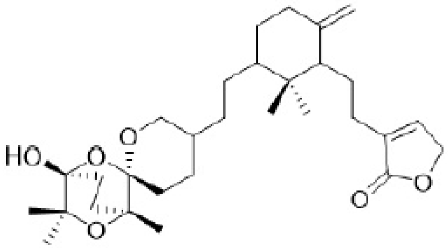

Saponaceolide Q

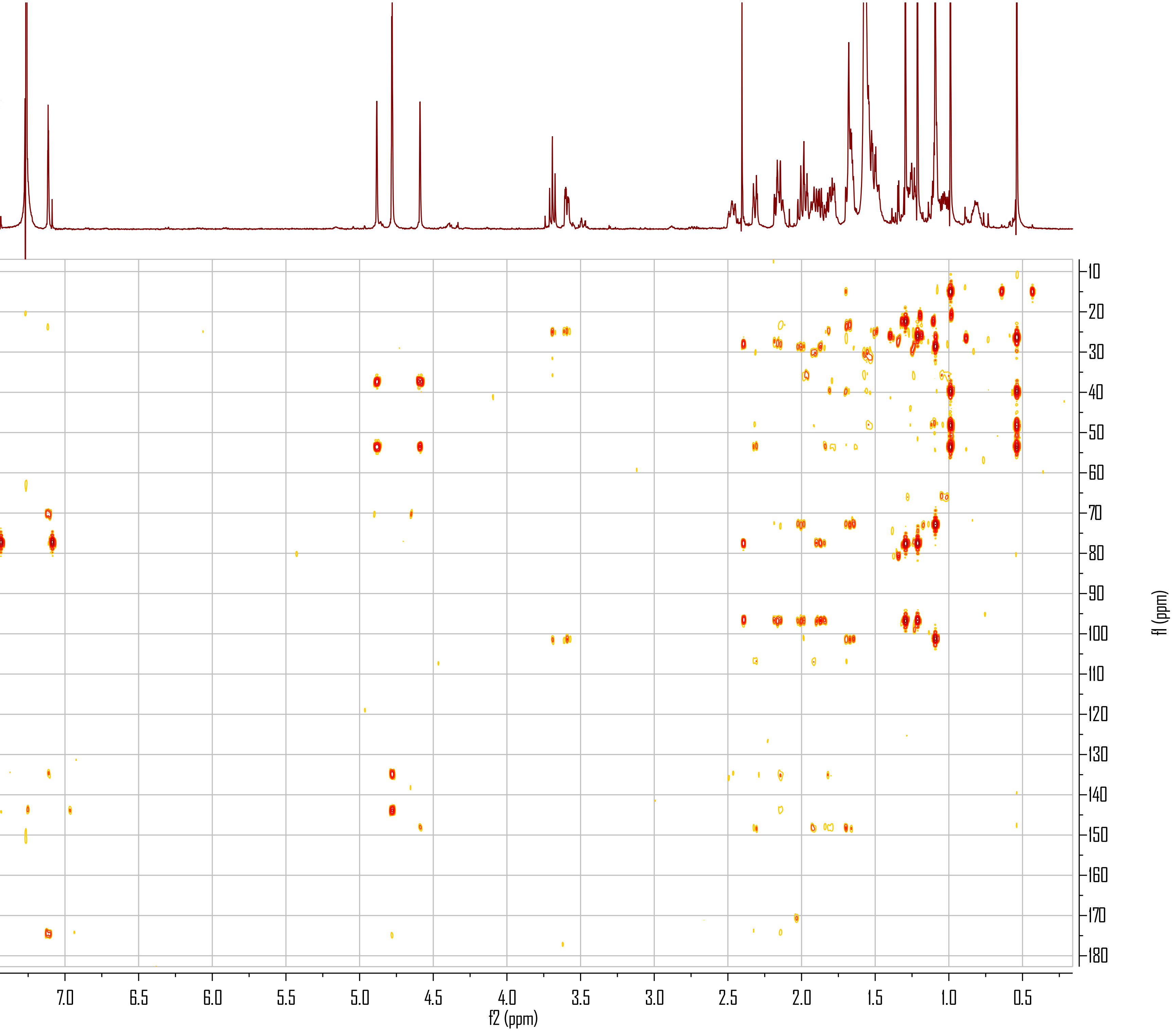

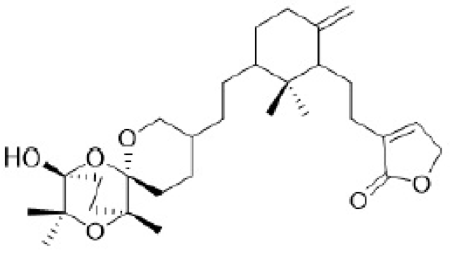
HO
O
O
O
O
O
Saponaceolide Q

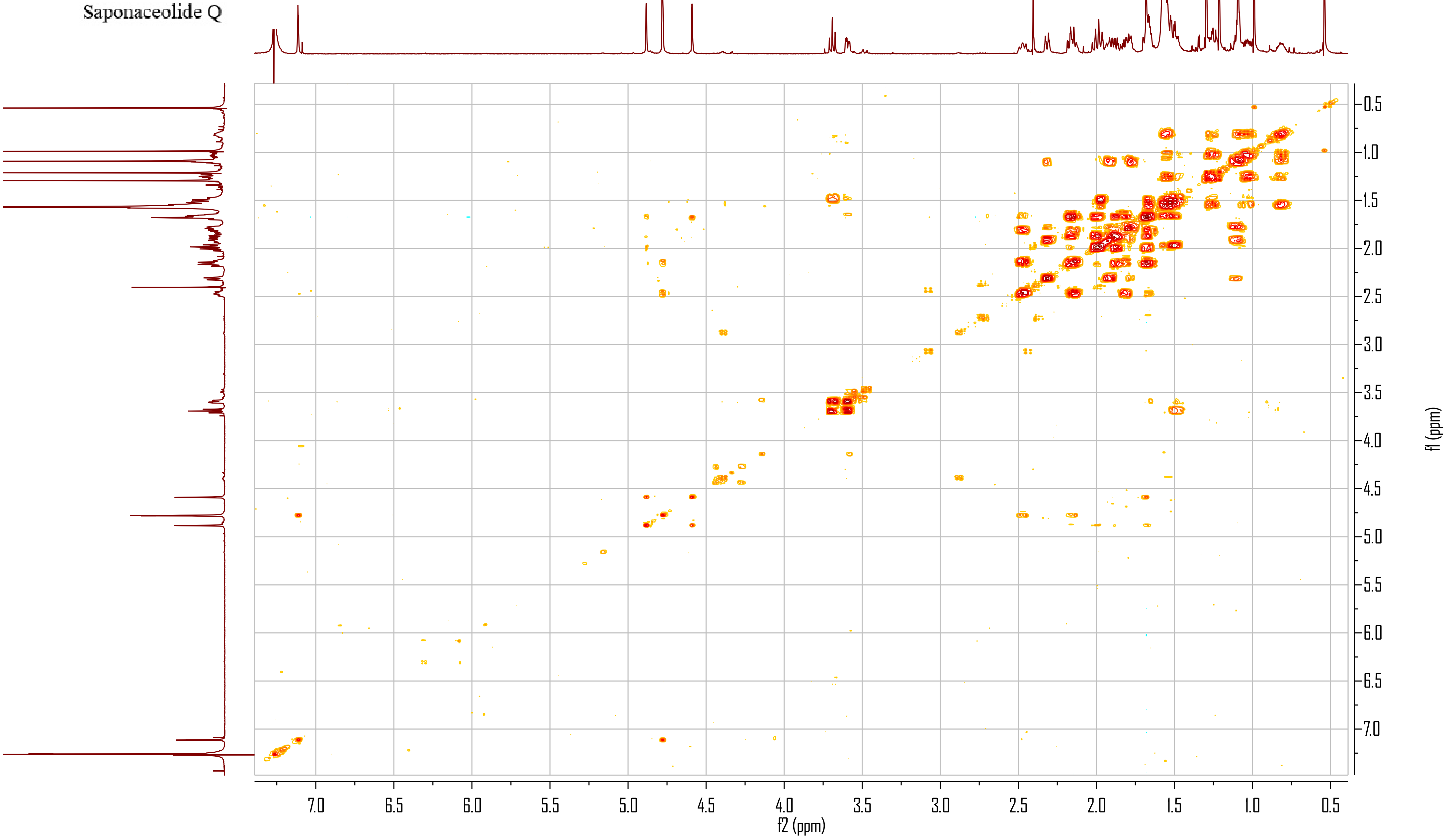
7.0
6.5
6.0
5.5
5.0
4.5
4.0
3.5
3.0
2.5
2.0
1.5
1.0
0.5
f2 (ppm)
f1 (ppm)

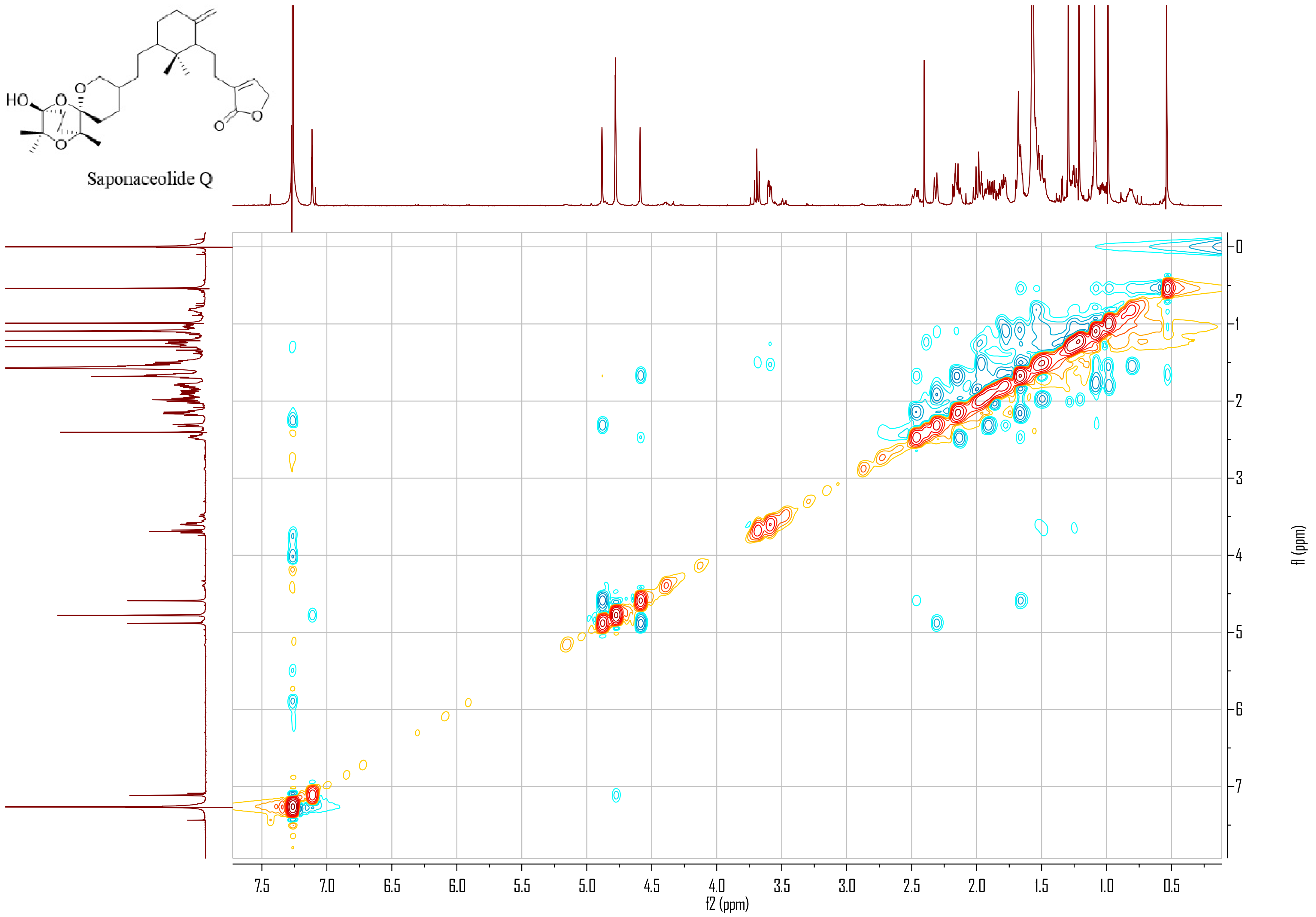

HO
O
O
O
O
O
Saponaceolide Q
f2 (ppm)
f1 (ppm)
7.5
7.0
6.5
6.0
5.5
5.0
4.5
4.0
3.5
3.0
2.5
2.0
1.5
1.0
0.5
0
1
2
3
4
5
6
7

Qualitative Analysis Report

**Data Filename**	lftp-34.d	**Sample Name**	lftp-34
**Sample Type**	Sample	**Position**	P1-D6
**Instrument Name**	Instrument 1	**User Name**	
**Acq Method**	SIBU.m	**Acquired Time**	7/24/2015 4:03:55 PM
**IRM Calibration Status**	Success	**DA Method**	Default.m
**Comment**			

**Sample Group**		**Info.**
**Acquisition SW Version**	6200 series TOF/6500 series Q-TOF B.05.01 (B5125.2)	

User Spectra

Fragmentor Voltage	Collision Energy	Ionization Mode
135	0	ESI

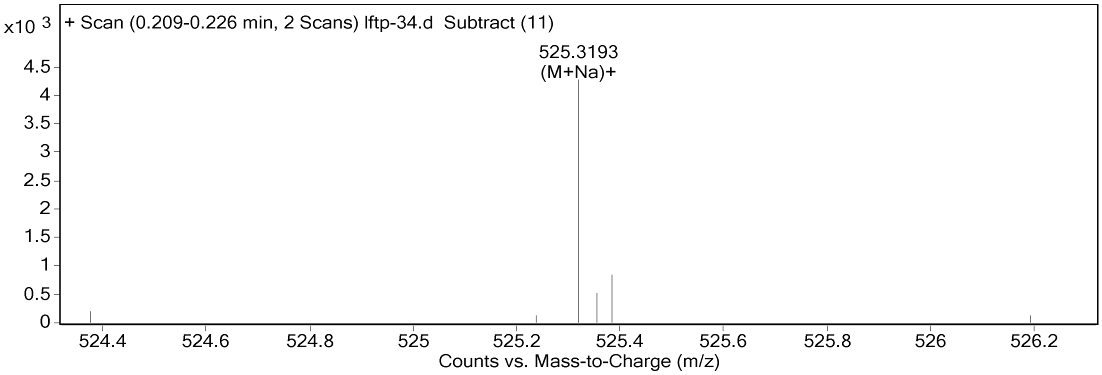

Peak List

m/z	z	Abund	Formula	Ion
286.1075	1	3827.21		
441.3726	1	5907.85		
457.3681	1	6943.4		
481.365	1	8299.9		
497.3573		8063.91		
511.3753	1	4852.8		
513.3414	1	4003.3		
525.3193	1	4266.81	C30 H46 O6	(M+Na)+

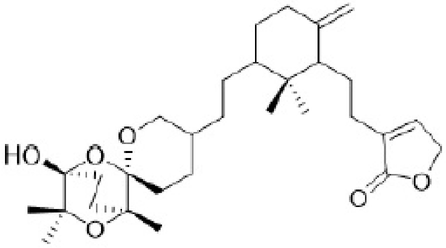

Saponaceolide Q

Formula Calculator Element Limits

Element	Min	Max
C	3	60
H	0	120
O	0	30

Formula Calculator Results

Formula	CalculatedMass	CalculatedMz	Mz	Diff. (mDa)	Diff. (ppm)	DBE
C30 H46 O6	502.3294	525.3187	525.3193	-0.4	-0.8	8.0000

--- End Of Report ---

NMR and HRESIMS for compound 2

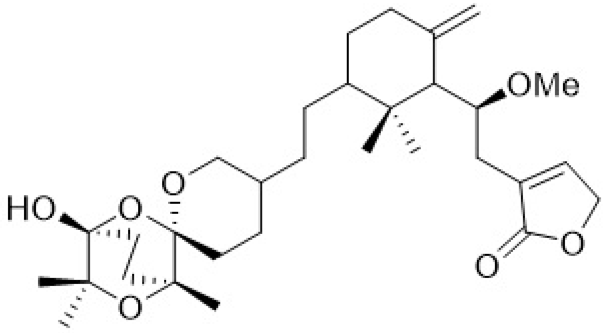

Saponaceolide R

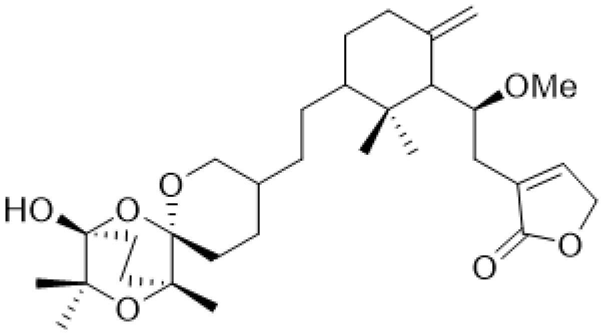

Saponaceolide R

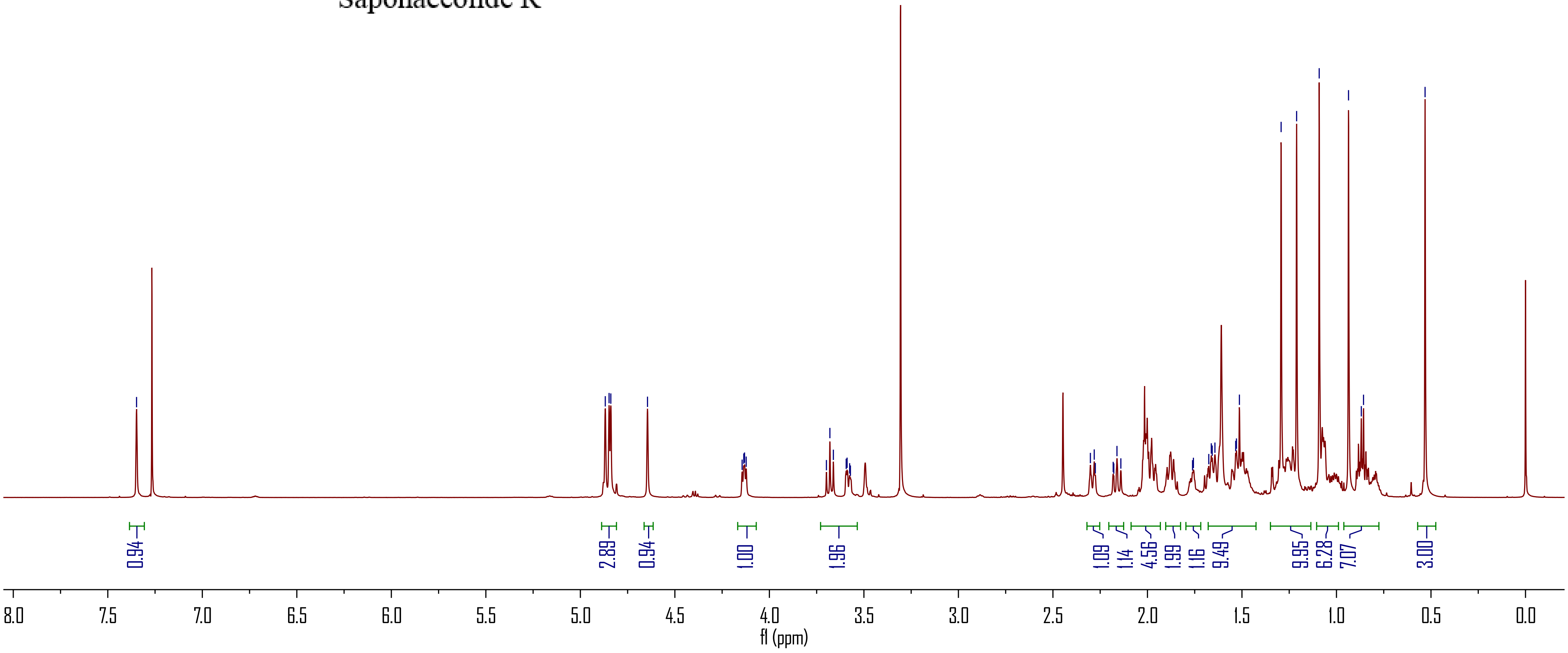

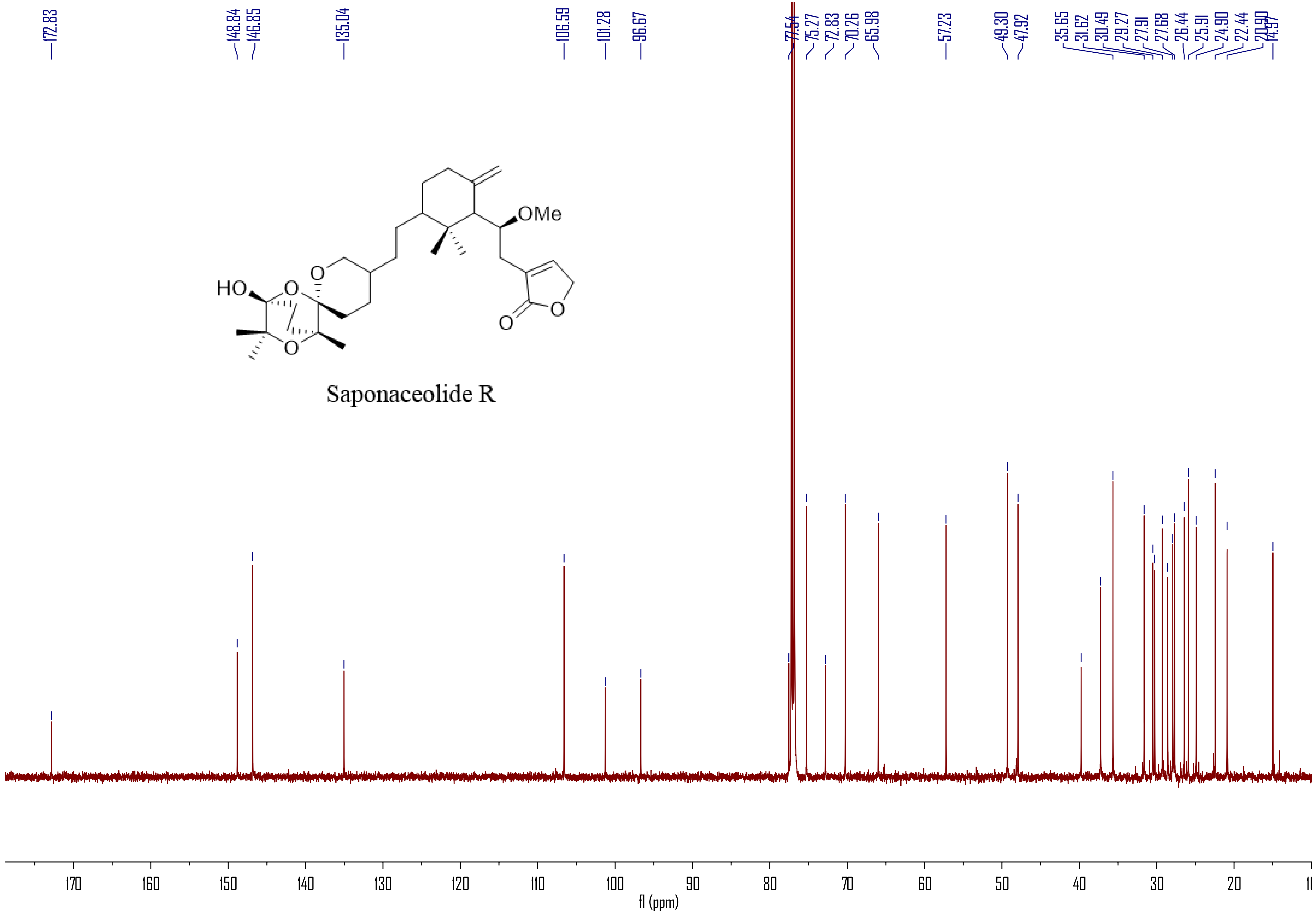
172.83
148.84
146.85
135.04
106.59
101.28
96.67
77.54
75.27
72.83
70.26
65.98
57.23
49.30
47.92
35.65
31.62
30.49
29.27
27.91
27.68
26.44
25.91
24.90
22.44
20.90
14.97
HO
O
O
O
OMe
O
O
Saponaceolide R
170
160
150
140
130
120
110
100
90
80
70
60
50
40
30
20
f1 (ppm)

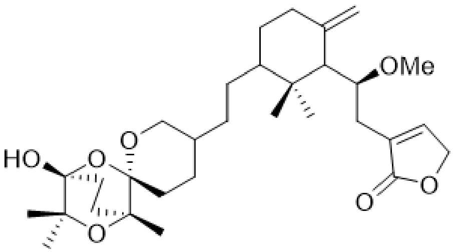

Saponaceolide R

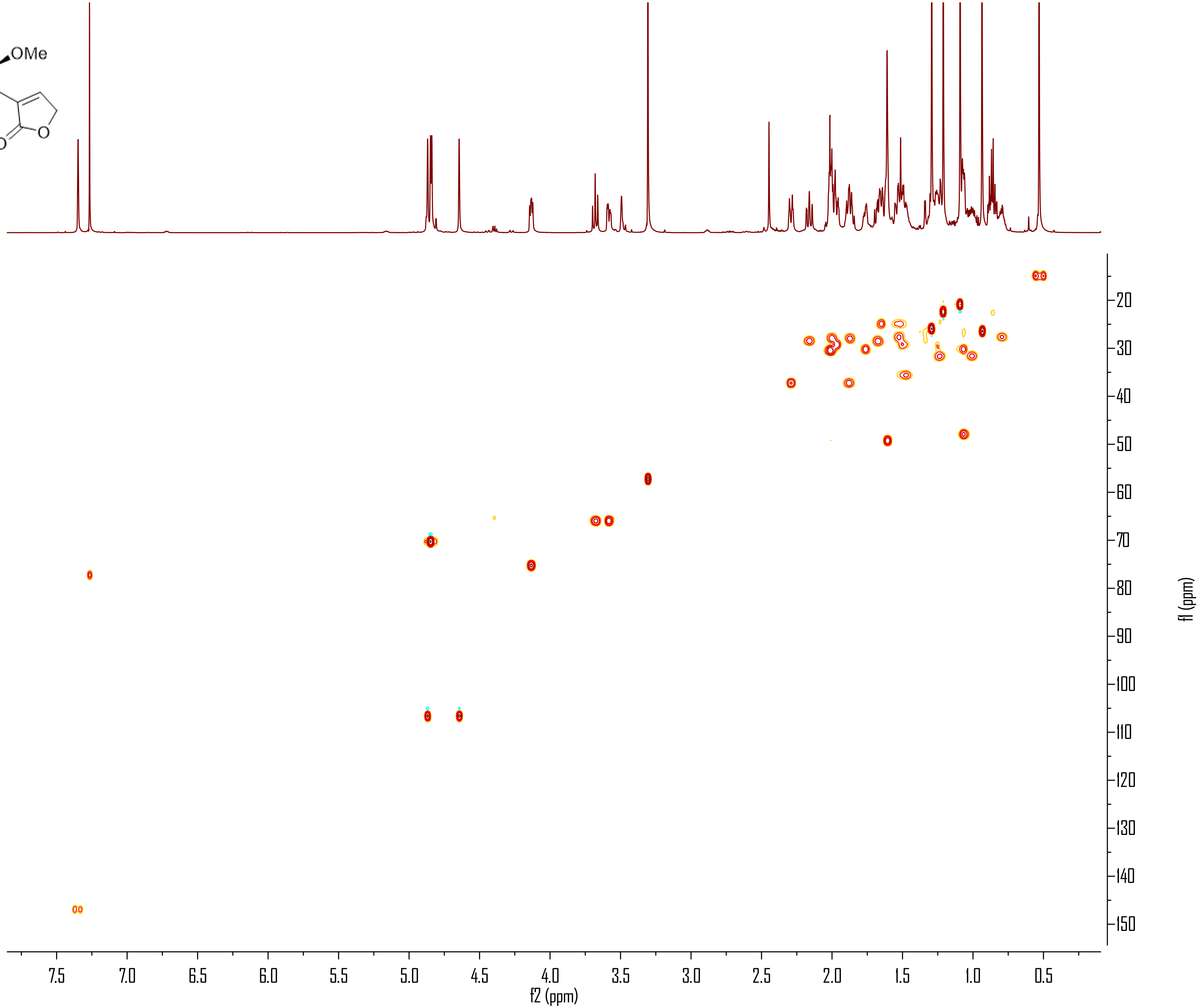

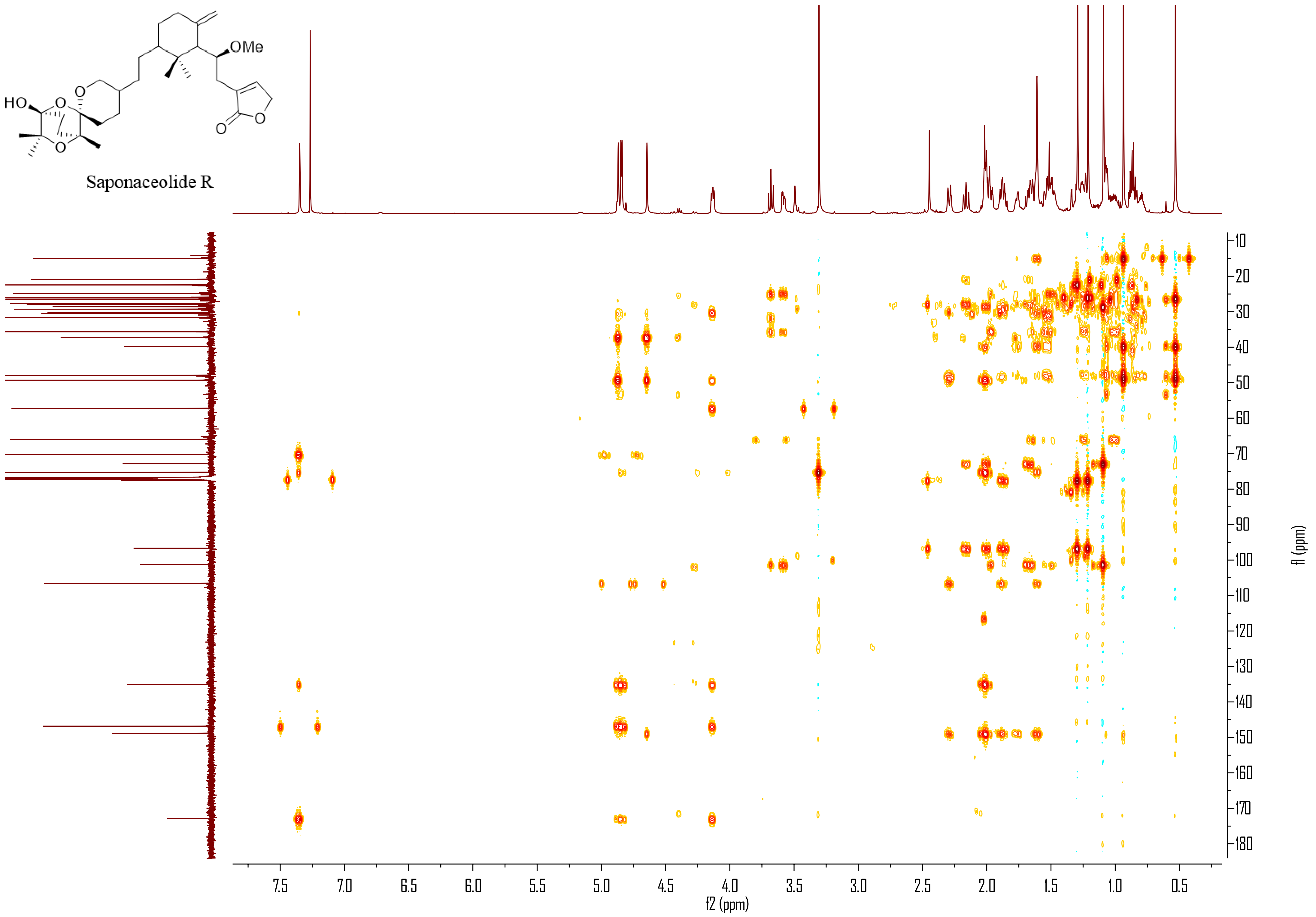

HO
O
O
O
OMe
O
O
Saponaceolide R
f2 (ppm)
f1 (ppm)

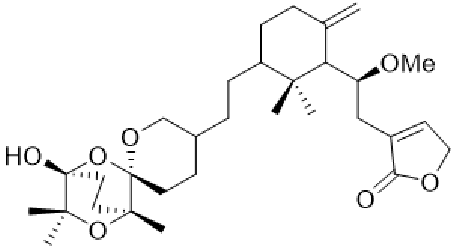

Saponaceolide R

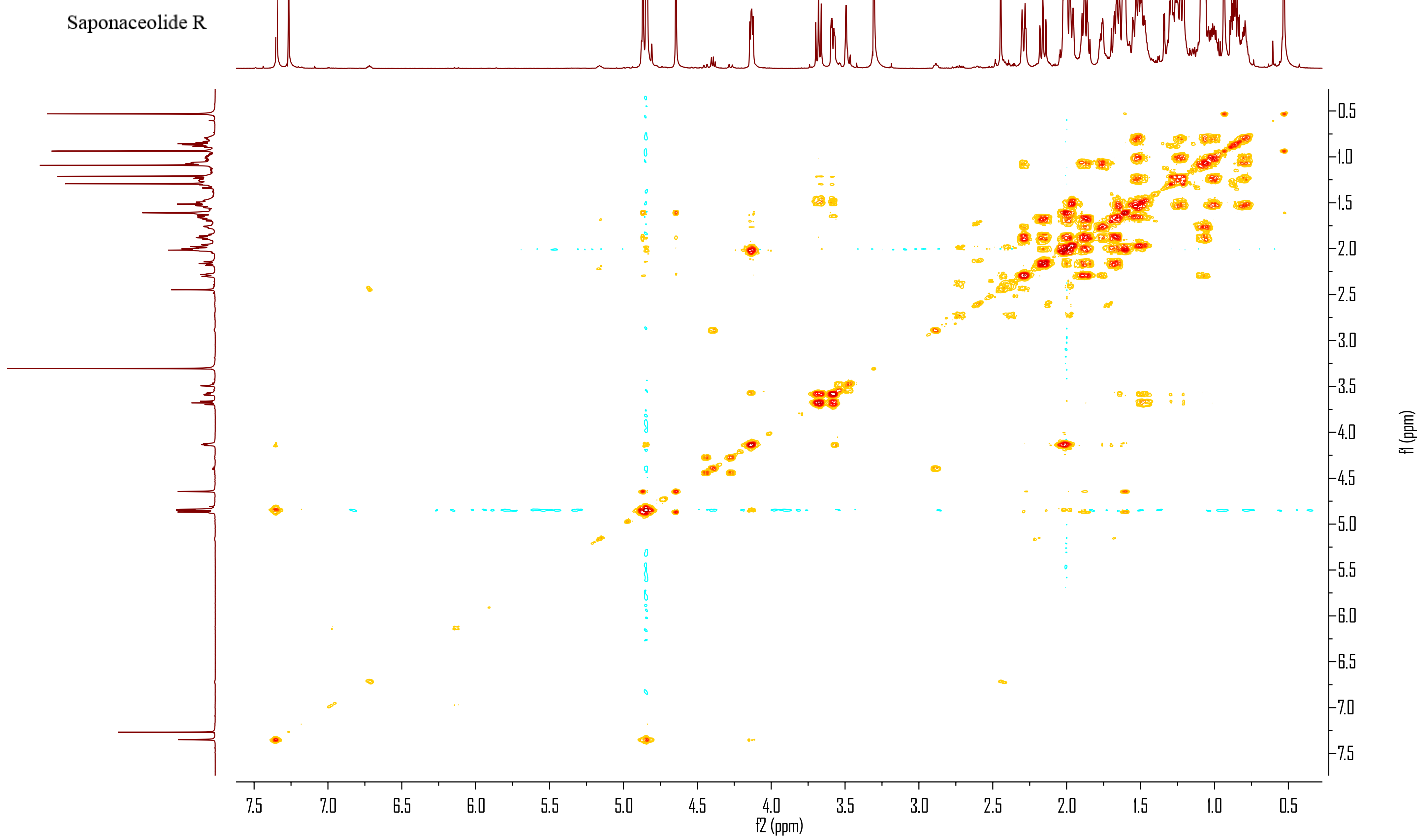

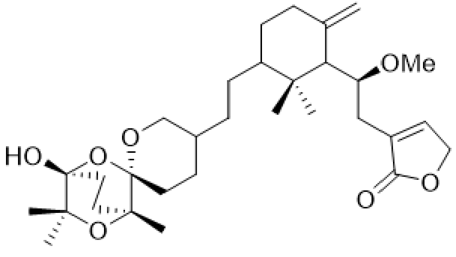

Saponaceolide R

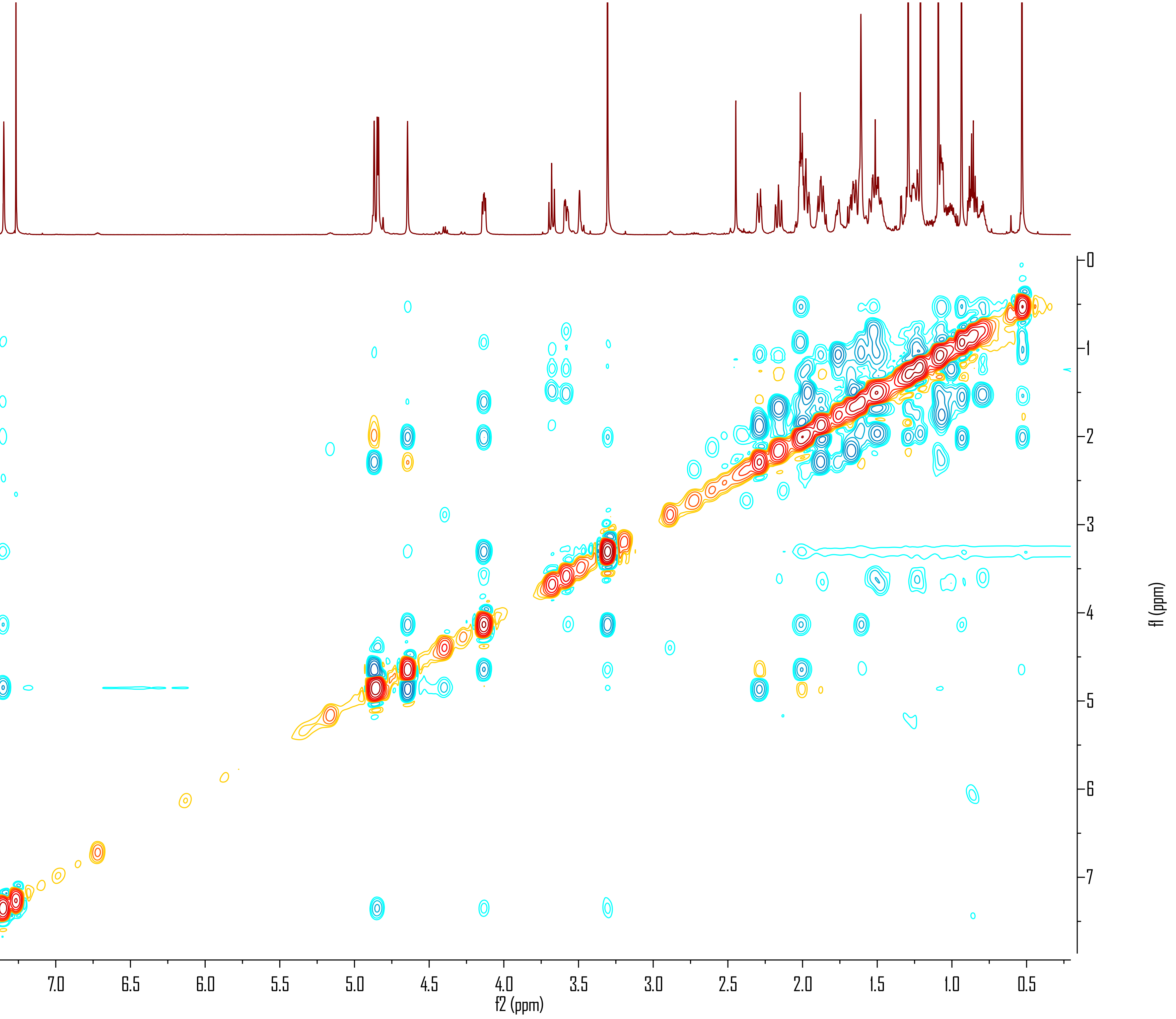

Qualitative Analysis Report

**Data Filename**	lftp-47.d	**Sample Name**	lftp-47
**Sample Type**	Sample	**Position**	P1-D8
**Instrument Name**	Instrument 1	**User Name**	
**Acq Method**	SIBU.m	**Acquired Time**	7/24/2015 4:06:55 PM
**IRM Calibration Status**	Success	**DA Method**	Default.m
**Comment**			

**Sample Group**		**Info.**
**Acquisition SW Version**	6200 series TOF/6500 series Q-TOF B.05.01 (B5125.2)	

User Spectra

Fragmentor Voltage	Collision Energy	Ionization Mode
135	0	ESI

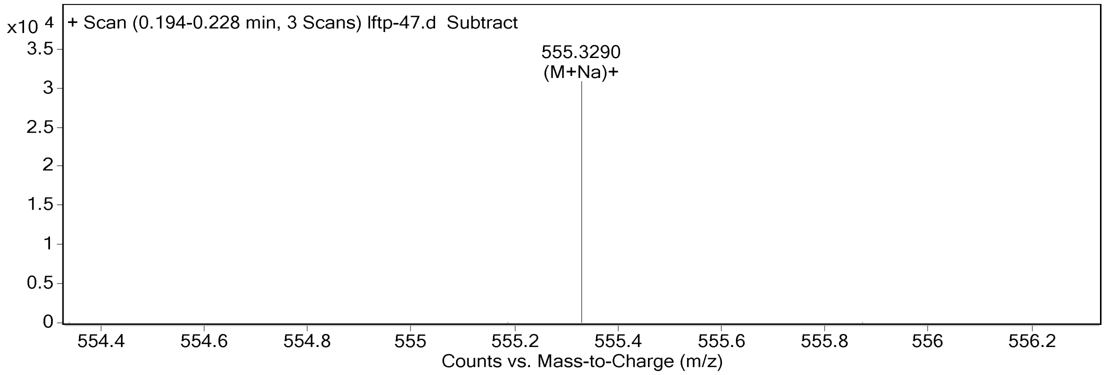

Peak List

m/z	z	Abund	Formula	Ion
274.2741	1	4904.99		
550.374	1	18301.29		
551.3773	1	6942.1		
555.329	1	30905.44	C31 H48 O7	(M+Na)+
556.3324	1	11328.69	C31 H48 O7	(M+Na)+
571.3033	1	20977.52		
572.3066	1	7397.35		
578.4044	1	4731.68		

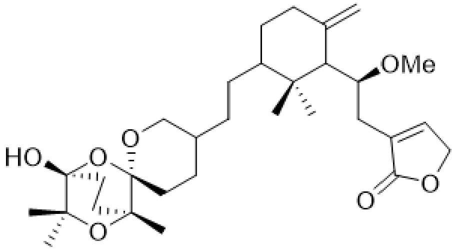

Saponaceolide R

Formula Calculator Element Limits

Element	Min	Max
C	3	60
H	0	120
O	0	20

Formula Calculator Results

Formula	CalculatedMass	CalculatedMz	Mz	Diff. (mDa)	Diff. (ppm)	DBE
C31 H48 O7	532.3400	555.3292	555.3290	0.4	0.7	8.0000

--- End Of Report ---

NMR and HRESIMS for compound 3

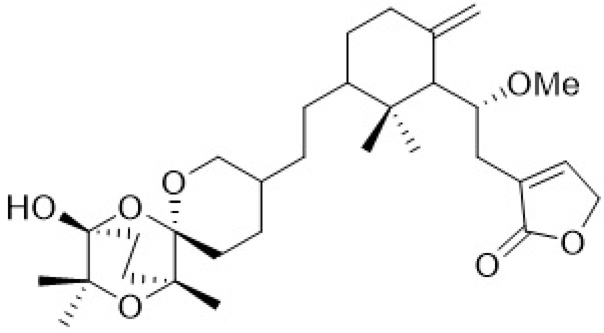

Saponaceolide S

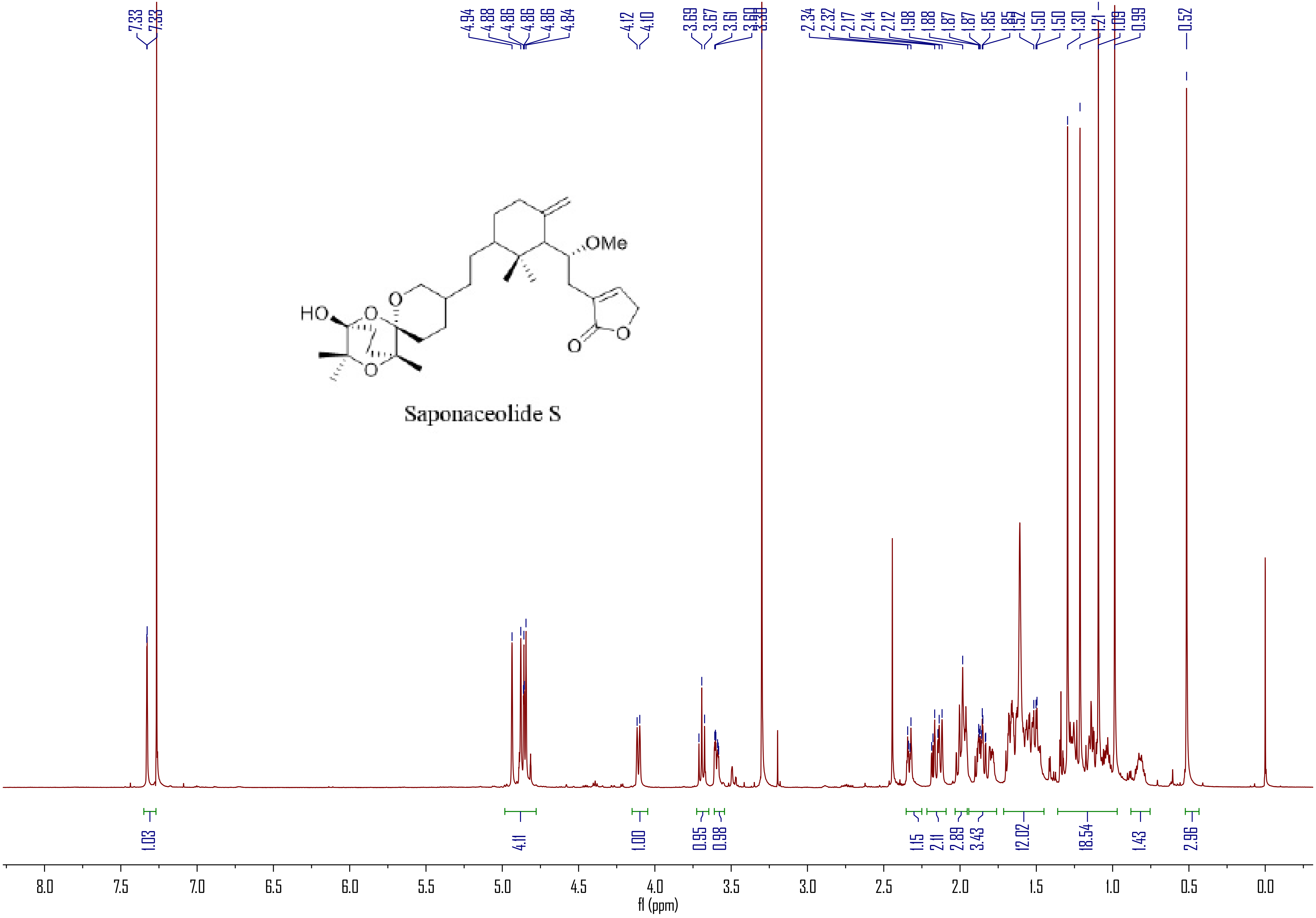

Saponaceolide S

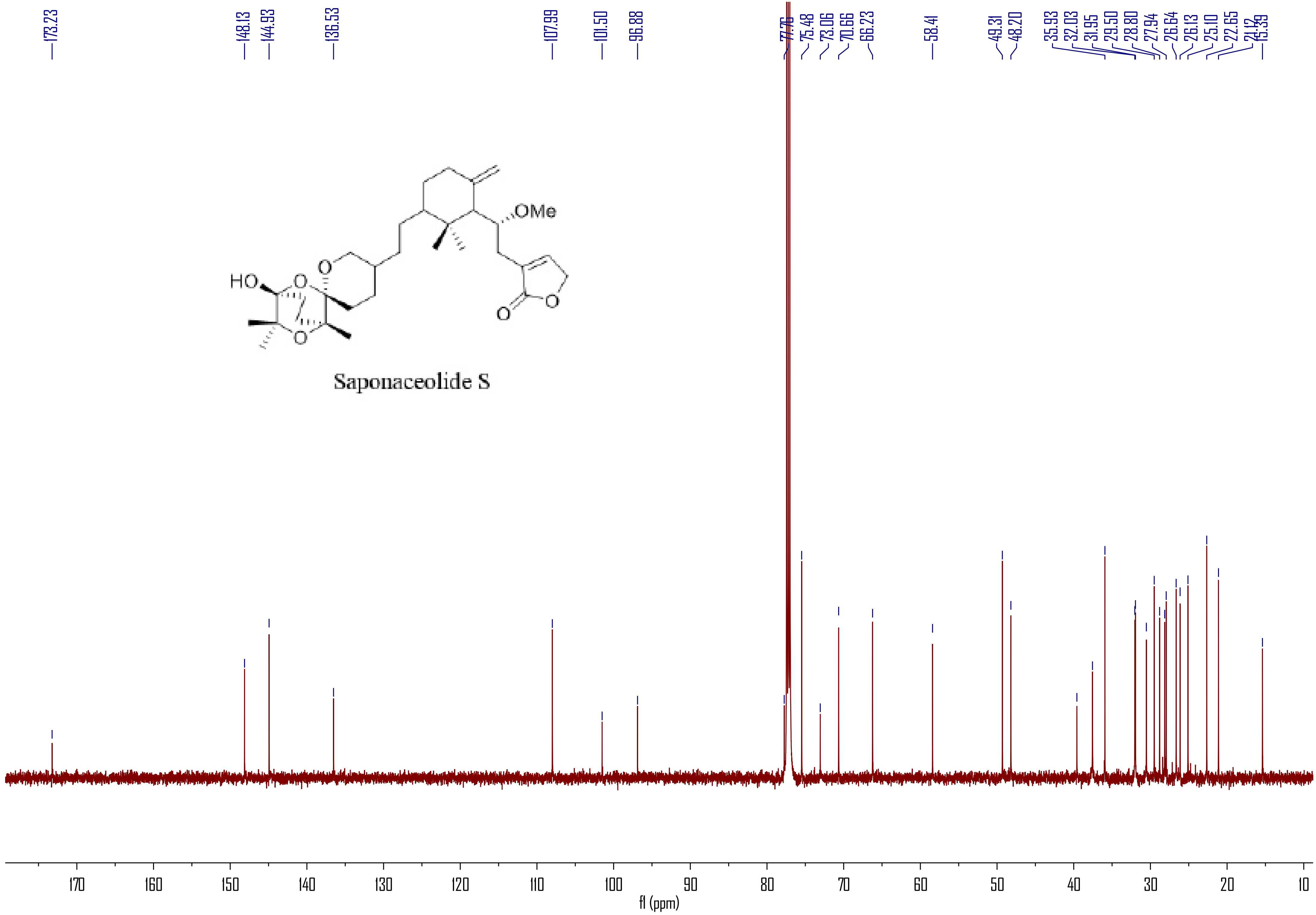

173.23
148.13
144.93
136.53
107.99
101.50
96.88
77.76
75.48
73.06
70.66
66.23
58.41
49.31
48.20
35.93
32.03
31.95
29.50
28.80
27.94
26.64
26.13
25.10
22.65
21.12
15.39
HO
O
O
O
OMe
O
O
Saponaceolide S
170
160
150
140
130
120
110
100
90
80
70
60
50
40
30
20
10
f1 (ppm)

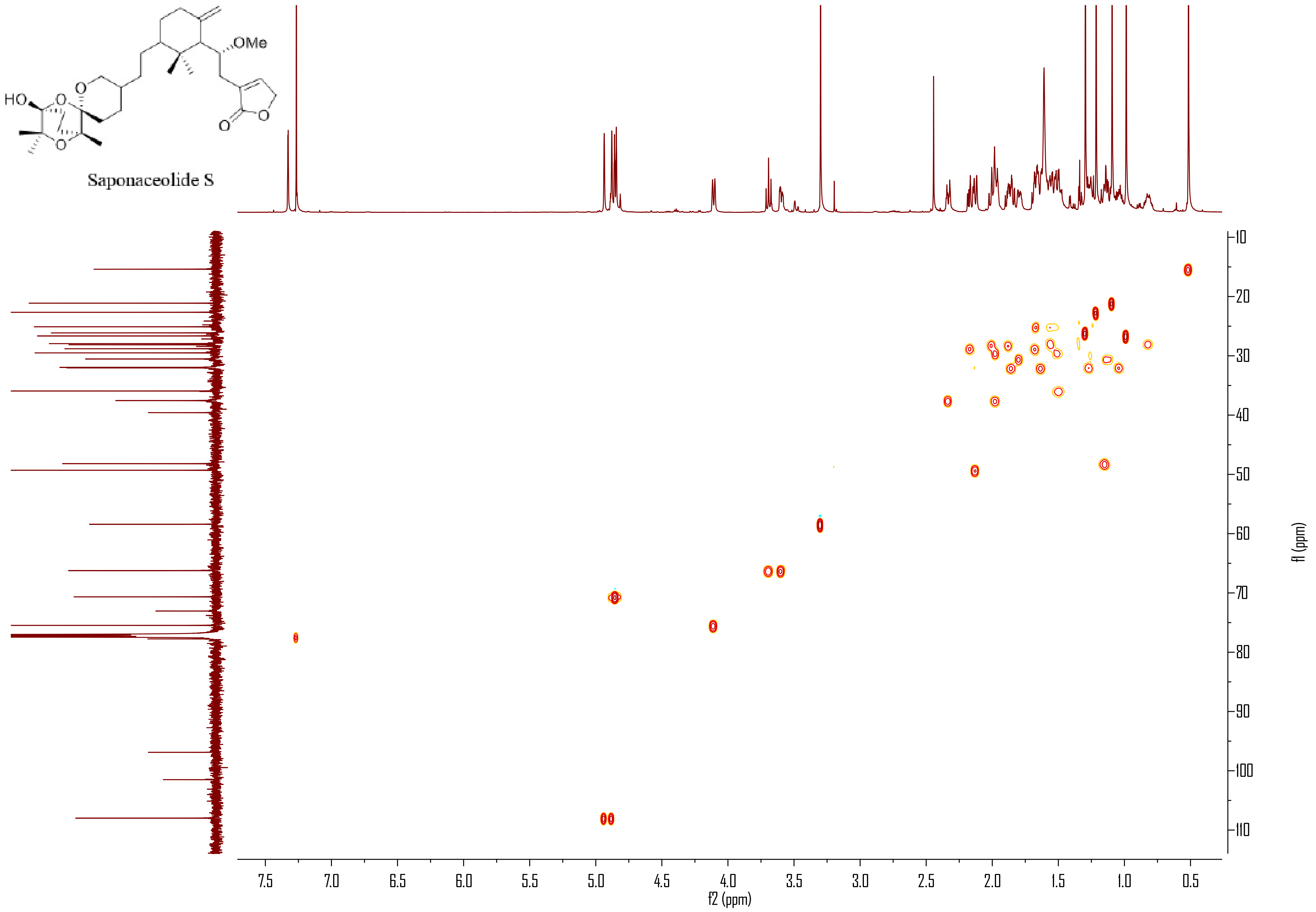
HO
O
O
O
OMe
O
O
Saponaceolide S
7.5
7.0
6.5
6.0
5.5
5.0
4.5
4.0
3.5
3.0
2.5
2.0
1.5
1.0
0.5
f2 (ppm)
10
20
30
40
50
60
70
80
90
100
110
f1 (ppm)

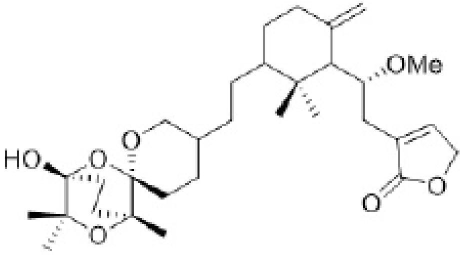

Saponaceolide S

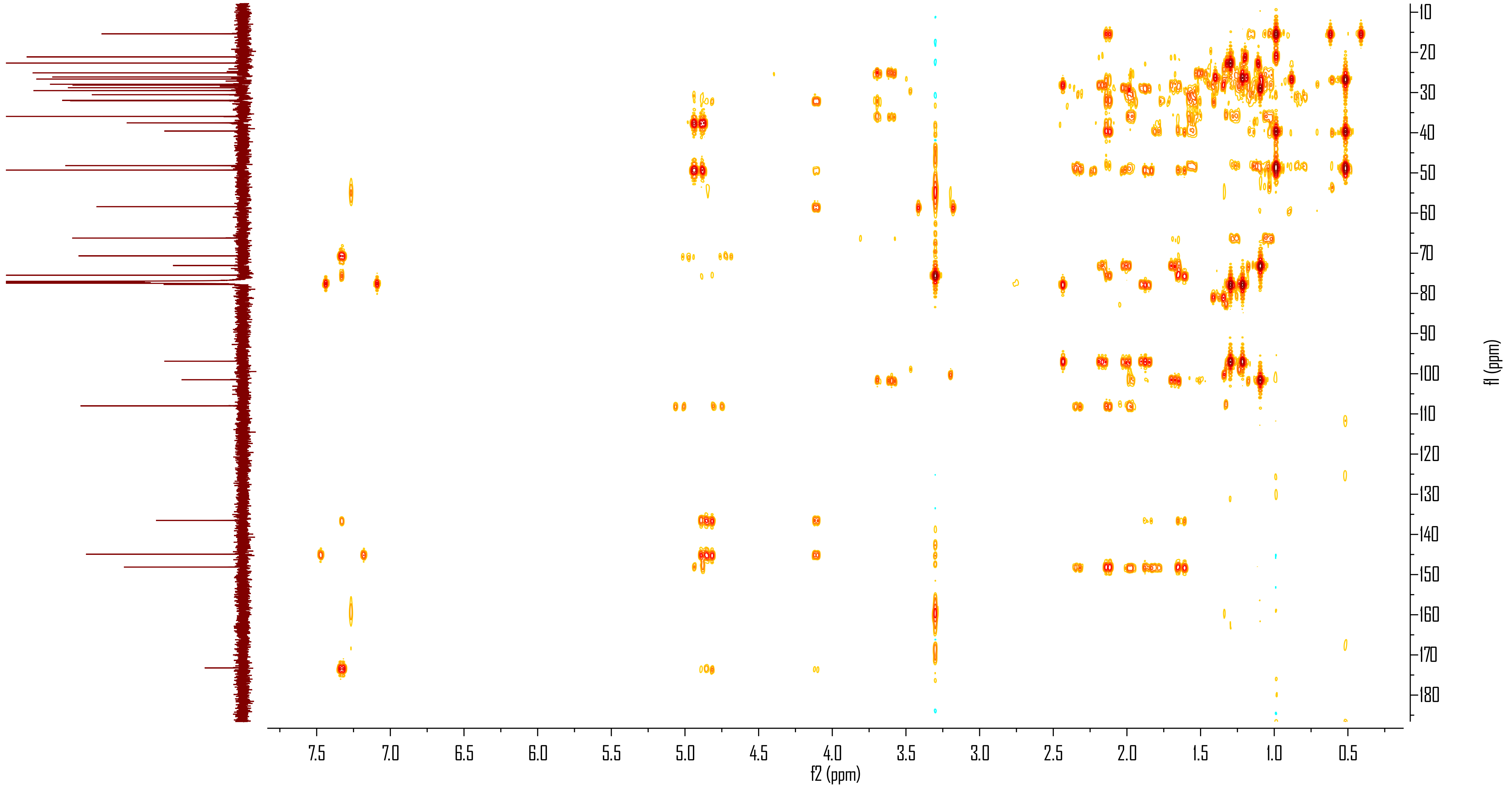

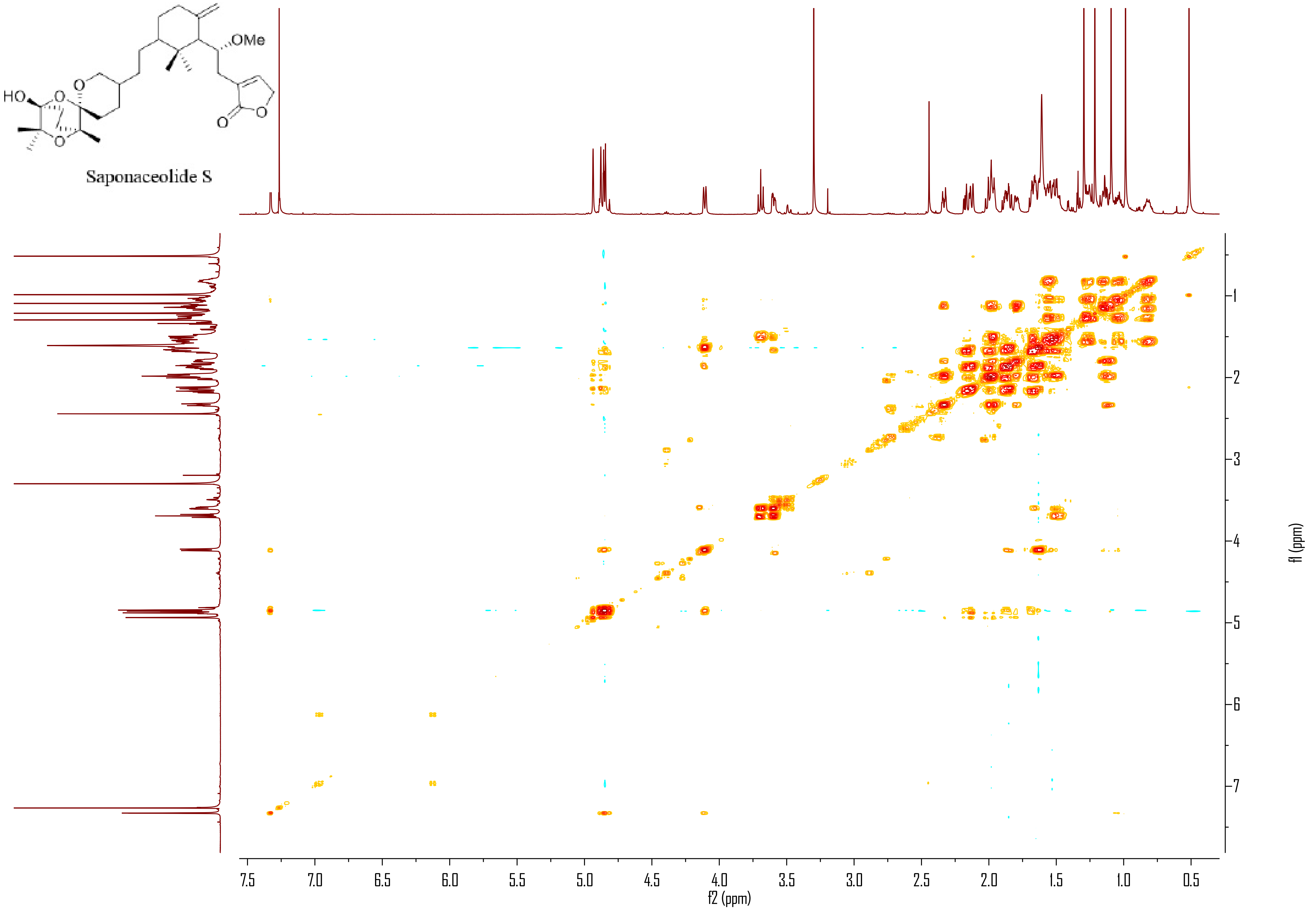

HO
OMe
Saponaceolide S
f1 (ppm)
f2 (ppm)

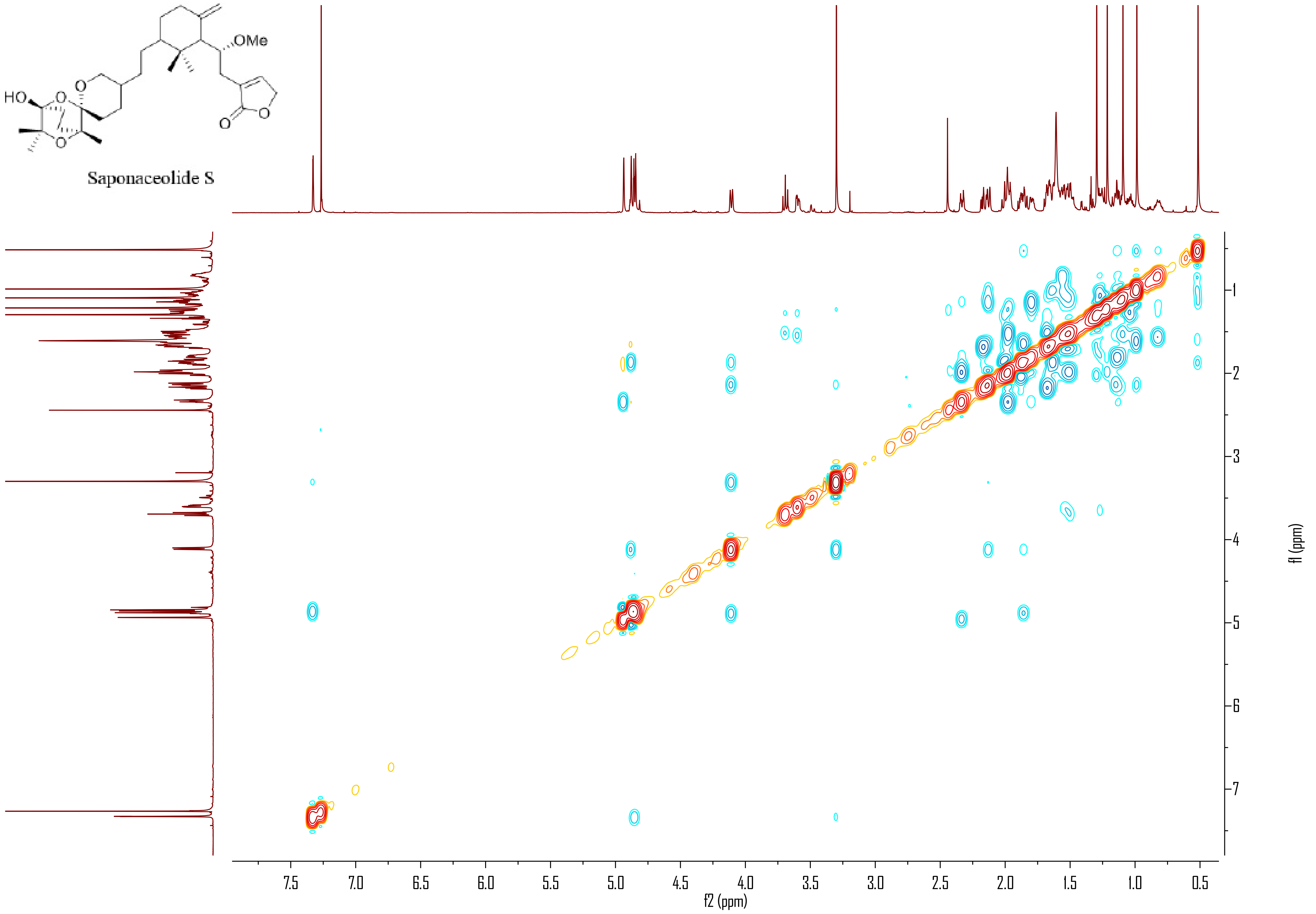

HO
O
O
O
OMe
O
O
Saponaceolide S
7.5
7.0
6.5
6.0
5.5
5.0
4.5
4.0
3.5
3.0
2.5
2.0
1.5
1.0
0.5
f2 (ppm)
1
2
3
4
5
6
7
f1 (ppm)

Qualitative Analysis Report

**Data Filename**	lftp-45.d	**Sample Name**	lftp-45
**Sample Type**	Sample	**Position**	P1-D7
**Instrument Name**	Instrument 1	**User Name**	
**Acq Method**	SIBU.m	**Acquired Time**	7/24/2015 4:05:25 PM
**IRM Calibration Status**	Success	**DA Method**	Default.m
**Comment**			

**Sample Group**		**Info.**
**Acquisition SW Version**	6200 series TOF/6500 series Q-TOF B.05.01 (B5125.2)	

User Spectra

Fragmentor Voltage	Collision Energy	Ionization Mode
135	0	ESI

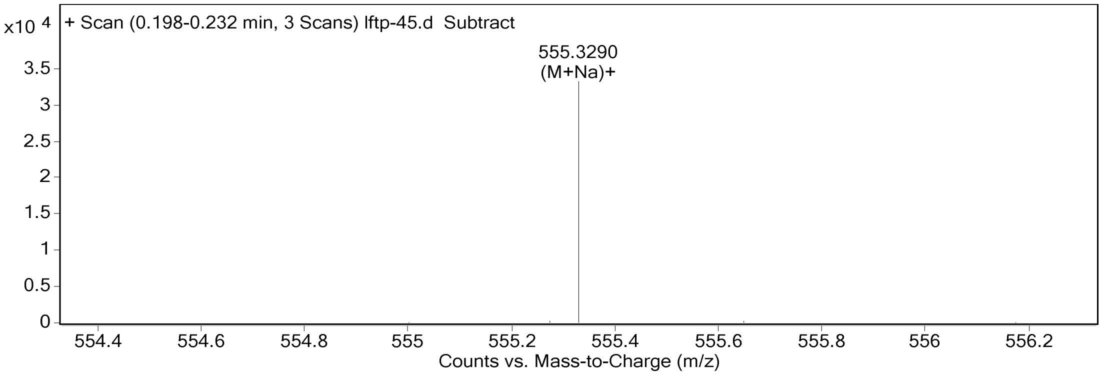

Peak List

m/z	z	Abund	Formula	Ion
274.2742	1	9727.79		
515.3366	1	7179.63		
550.3741	1	18770.78		
555.329	1	33298.01	C31 H48 O7	(M+Na)+
556.3329	1	11434.78	C31 H48 O7	(M+Na)+
571.3027	1	24889.4		
572.3063	1	8635.41		
578.4048	1	7795.31		

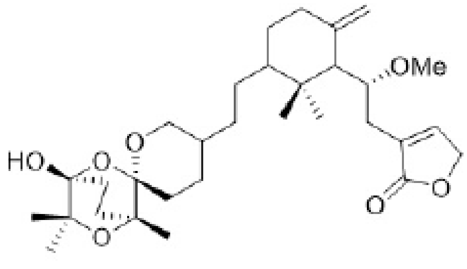

Saponaceolide S

Formula Calculator Element Limits

Element	Min	Max
C	3	60
H	0	120
O	0	20

Formula Calculator Results

Formula	CalculatedMass	CalculatedMz	Mz	Diff. (mDa)	Diff. (ppm)	DBE
C31 H48 O7	532.3400	555.3292	555.3290	0.3	0.6	8.0000

--- End Of Report ---
